# Splenic hematoma may present as large bowel obstruction: A case report

**DOI:** 10.1016/j.ijscr.2018.11.012

**Published:** 2018-11-22

**Authors:** Christine Kolwitz, Christopher Esposito, Caitlin Gauvin, Vinaya Gaduputi, Brian Chiong, Tagore Sunkara, Gerard A. Baltazar

**Affiliations:** a Division of Trauma, Bronx, NY, USA; b Division of Gastroenterology and Hepatology, Bronx, NY, USA; c Department of Radiology SBH Health System, Bronx, NY, USA; d School of Medicine, City University of New York, New York, NY, USA; e College of Osteopathic Medicine, New York Institute of Technology, Old Westbury, NY, USA

**Keywords:** Trauma, Spleen, Colon, Bowel obstruction, Case report

## Abstract

**Introduction:**

Large bowel obstruction (LBO) warrants prompt evaluation and management. Although causes of LBO are most commonly intrinsic to the colon (e.g. malignancy, diverticular stricture, intussusception or volvulus), rare extrinsic etiologies exist. An extremely rare extrinsic etiology of LBO described only once, is compressive splenic hematoma.

**Presentation of case:**

A 64-year-old female presented to the emergency department complaining of two days of diffuse abdominal pain and distension, watery diarrhea and nausea subsequent to a mechanical fall to her left side.

Computed tomography demonstrated a grade 3 splenic hematoma with active extravasation, causing extrinsic compression and obstruction of the colon. Embolization of the splenic artery was performed, and non-operative LBO management resulted in resumption of normal bowel function after six days.

**Discussion:**

To our knowledge, the only other case of colonic compression by splenic hematoma (a case report in the radiology literature from 1994) describes a 62-year-old male whose symptoms similarly spontaneously resolved. Increasing frequency of non-operative management of splenic trauma may result in increased frequency of splenic hematoma complications. Physicians and surgeons who treat LBO should be aware of this rare etiology and its potential for non-operative management.

**Conclusion:**

Our case demonstrates the importance of considering splenic hematoma as an etiology of LBO, particularly in the setting of trauma and that management of this entity can be successfully non-operatively.

## Highlights

• Traumatic splenic hematoma may present as large bowel obstruction.
• Large bowel obstruction secondary to splenic hematoma may become a more-frequently recognized phenomenon.
• Large bowel obstruction secondary to splenic hematoma may be managed non-operatively and resolve in approximately one week.

## 1 Introduction

Etiologies of large bowel obstruction (LBO) are commonly intrinsic to the colon with malignancy accounting for up to 85% of cases [1]. Less common intrinsic causes include diverticular or ischemic stricture, intussusception and volvulus [1]. Extrinsic etiologies of LBO are exceedingly rare.

Prompt evaluation and management of LBO are essential, and delays may result in poor outcomes, including dehydration, critical electrolyte aberrancies, bowel perforation and death [[1], [2], [3]]. Urgent surgical management of LBO is required in cases of bowel perforation or colonic vascular compromise [1,3]; however, when evidence of such sequelae is not present, non- operative management may be pursued.

Management of splenic trauma is increasingly non-operative (i.e. selective non-operative management, SNOM), and a unique array of complications may result from splenic SNOM [4]. Gastrointestinal complications occur among 3–16% of patients undergoing SNOM for splenic injury, significantly more frequently if splenic artery embolization (SAE) is employed [4].

Herein, we present a case of extrinsic LBO caused by splenic hematoma after a fall. To our knowledge, this is only the second such case described. This work has been reported in line with the SCARE criteria [5].

## 2 Presentation of case

A 64-year-old female with past medical history of hemorrhagic stroke status-post ventriculo- peritoneal shunt and brain aneurysm clipping, seizures and multiple episodes of small bowel obstructions and exploratory laparotomies with lysis of adhesions presented by ambulance to the emergency department of an urban university-affiliated community-based trauma center, complaining of diffuse abdominal pain, distension and pressure for two days. She endorsed having watery diarrhea and nausea and no flatus for one day but denied other systemic complaints. She ambulates with an assistive device and had a fall to her left side in proximity to the start of her abdominal symptoms.

On physical exam, her abdomen was tender diffusely but non-peritoneal and profoundly distended with hypoactive bowel sounds and increased tympany.

**Fig. 1:**
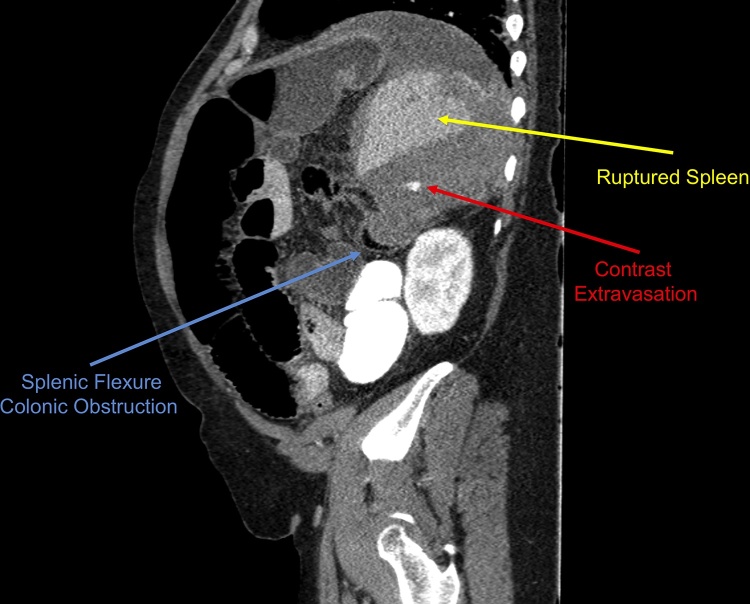
Post-traumatic splenic hematoma with active contrast extravasation causes extrinsic compression of splenic flexure, resulting in large bowel obstruction. Computed tomography coronal sagittal section on initial presentation.

Computed tomography (CT) of the abdomen and pelvis demonstrated a grade 3 splenic hematoma with evidence of active extravasation and intra-abdominal fluid consistent with hemorrhage. The CT also revealed extrinsic colonic compression by the splenic hematoma which was causing a large bowel obstruction (LBO)—the obstruction had decompressed through the incompetent ileocecal valve with resultant small bowel dilatation (Fig. 1).

Initial management consisted of non-operative measures as there was no evidence of hemodynamic instability, peritonitis or high risk of bowel perforation. Splenic arterial embolization (SAE) was completed with five 5 mm coils and six 2 mm coils followed by gelfoam alurry with approximately 90% splenic devascularization.

The patient was subsequently admitted to the surgical intensive care unit for serial abdominal exams, abdominal x-rays and hematocrit level evaluations. She was made *nil per os*. She

received a total two units packed red blood cells during admission. We administered mineral oil enemas in order to encourage fecal motility.

CT of the abdomen and pelvis conducted three days post-SAE demonstrated diffuse hemoperitoneum, larger mesenteric-peritoneal hematoma but no significant change in the splenic hematoma—importantly, a small amount of colonic contrast appeared to migrate past the splenic hematoma.

**Fig. 2:**
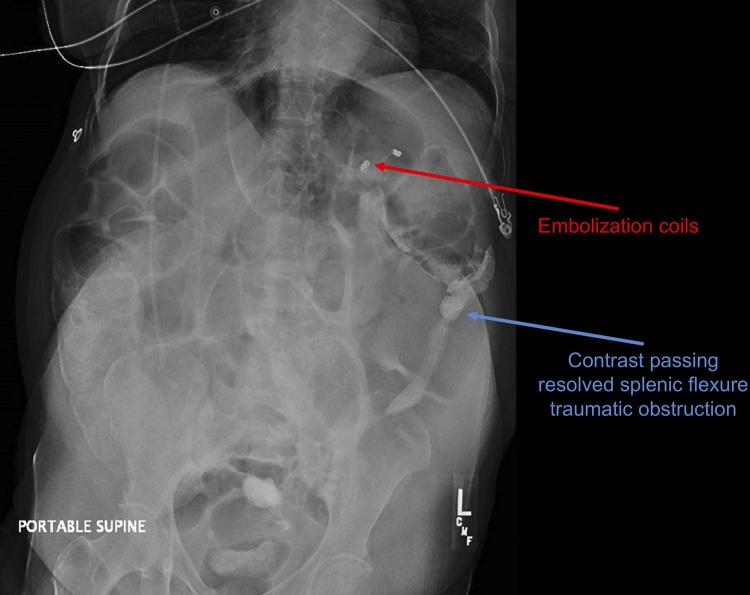
Abdominal x-ray reveals contrast migrating past the area of the splenic hematoma on post-splenic artery embolization day six.

Oral diet was advanced as the patient’s bowel function returned to normal. Abdominal x-ray on post-SAE day six revealed contrast clearly migrating past the area of extrinsic compression consistent with report of normalized bowel function (Fig. 2).

The patient was discharged home in stable condition after post-SAE vaccinations had been administered. At two-week follow-up, she required no further surgical intervention.

## 3 Discussion

In 1994,Whittick and Viamonte described the first (and only) case of colonic obstruction caused by splenic hematoma [6]. We present the second case of LBO caused by splenic hematoma.

Similar to the previous case, our patient is a sexagenarian who suffered a fall in proximity to the onset of abdominal pain and distension. Imaging revealed similar obstructive features; however, active extravasation of contrast was unique to our case and necessitated SAE. The SNOM hospital course was similar to the previously-reported management and appropriate in the setting of hemodynamic stability and absence of peritonitis.

We opted to use mineral oil enemas to facilitate bowel movements under the assumption that solid stools proximal to the obstruction would be softened. Although this treatment appeared to produce an early bowel movement, the utility in the setting of extrinsic LBO remains unclear.

The length of time necessary to allow return of colonic function is likewise unclear—the 1994 case resolved within a week, similar to the presented timeline, and we suggest grossly exceeding this time-frame represents failure of SNOM, presuming splenic hemostasis. We recommend consideration of invasive intervention (e.g. parenteral nutrition, colonic stenting or splenectomy) after this time period or if patient significantly clinically worsens.

Close follow-up is necessary for any splenic hematoma including post-SAE as bleeding may recur or abscess may develop. Freitas et al describe several complications that may occur following SNOM of blunt splenic injury, including 3–16% gastrointestinal (GI) complications (as defined by Kassin et al.) [4,7]. Although a significantly higher rate of GI complications after SNOM is expected post-SAE, both patients with LBO secondary to splenic hematoma had not

undergone SAE before developing GI symptoms. Additional cases would have to be analyzed to determine the actual risk of developing LBO during SNOM for splenic injury.

Our patient remained hemodynamically-stable and without peritonitis throughout her hospital course and also in the outpatient setting. Overall, the natural history of LBO caused by splenic hematoma seems relatively benign.

## 4 Conclusion

We present the second case of LBO caused by extrinsic compression by traumatic splenic hematoma. Our case demonstrates the importance of considering splenic hematoma as an etiology of LBO, particularly in the setting of trauma. The management of this entity can be non- operative if splenic hemostasis has been achieved and in the absence of peritonitis and will likely resolve within one week.

## Conflicts of interest

The authors (CK, CE, VG, BC, TS & GAB) declare no conflicts of interests or disclosures.

## Sources of funding

This work received no funding.

## Ethical approval

Exemption from IRB approval was provided by the SBH Health System Institutional Review Board.

## Consent

Written informed consent was obtained from the patient for publication of this case report and accompanying images. A copy of the written consent is available for review by the Editor-in-Chief of this journal on request.

## Author’s contribution

GAB is the primary investigator and contributed to conceptualization, data curation, formal analysis, project administration, supervision and writing and editing of the manuscript.

CK is the primary author and contributed to conceptualization, data curation and writing of the manuscript.

CE is the secondary author and contributed to data curation and writing of the manuscript.

VG, BC and TS contributed to data curation, validation, visualization and writing and editing of the manuscript.

## Registration of research studies

This does not seem necessary for Case Reports that are not first-in-man.

## Guarantor

Gerard A. Baltazar DO FACOS is the Guarantor of this work.

## SCARE

This work complies with SCARE guidelines.

## Provenance and peer review

Not commissioned, externally peer reviewed.
